# Enhanced recovery and reduced opioid requirements following robot-assisted minimally invasive gastrectomy: a retrospective cohort study

**DOI:** 10.1007/s11701-025-02839-8

**Published:** 2025-10-02

**Authors:** Katrin Winter, Nader El-Sourani, Carsten Szardenings, Ann-Kathrin Eichelmann, Jennifer Merten, Andreas Pascher, Mazen A. Juratli, Jens Peter Hoelzen

**Affiliations:** 1 Department of General, Visceral and Transplant Surgery, University Hospital Muenster, Albert-Schweitzer-Campus 1, 48149 Muenster, Germany; 2 Institute of Biostatistics and Clinical Research, University of Muenster, Muenster, Germany

**Keywords:** Gastrectomy, Gastric cancer, Opioid consumption, Postoperative pain, Retrospective cohort study, Robot-assisted surgery

## Abstract

Gastric cancer requires surgical resection for cure, with robot-assisted minimally invasive gastrectomy (RAMIG) emerging as an alternative to open gastrectomy (OG). Comparative data on postoperative pain and recovery remain limited. This study aimed to compare RAMIG versus OG in patients with resectable gastric cancer, focusing on postoperative opioid consumption, pain intensity, and recovery parameters. In this retrospective cohort study, 138 patients with resectable gastric cancer underwent either RAMIG (*n* = 39) or OG (*n* = 99) between May 2021 and August 2023. Primary endpoints were pain intensity (Numerical Rating Scale (NRS)) and opioid consumption. Secondary endpoints comprised intensive/intermediate care (ICU/IMC) and hospital stays, blood loss, severe complications, and operative duration. Statistical analysis used SPSS version 29.0 with Mann–Whitney *U* and Fisher’s exact tests (*p* < 0.05). RAMIG showed reduced opioid consumption (*p* = 0.002) and lower NRS scores during mobilization on days 5 and 7 (*p* = 0.011; *p* = 0.002) and at rest on day 7 (*p* = 0.005). The RAMIG group experienced significantly shortened ICU/IMC stays (*p* < 0.001), reduced hospitalization duration (*p* < 0.001), and decreased intraoperative blood loss, although operative duration was prolonged. RAMIG demonstrates favorable outcomes regarding opioid requirements, pain management, ICU/IMC and hospital stays, and blood loss compared to OG, despite longer operative duration. These findings support RAMIG as an effective approach enabling accelerated recovery in patient-centered care, though prospective randomized validation studies are warranted. *Trial registration*: DRKS00036368, retrospectively registered 11th of March 2025.

**Supplementary Information:**

The online version contains supplementary material available at 10.1007/s11701-025-02839-8.

## Introduction

Gastric cancer represents a significant global health burden, with surgical resection and D2 lymphadenectomy remaining the cornerstone of curative treatment for locally advanced diseases without distant metastasis [1–3].

Although laparoscopic approaches represent a well-accepted minimally invasive alternative [4, 5], the multicenter LOGICA trial demonstrated comparable outcomes between laparoscopic and open gastrectomy (OG) in Western patients, highlighting the need to evaluate whether robotic assistance provides measurable advantages over conventional open surgery [6].

Laparoscopic gastrectomy is limited by two-dimensional visualization, restricted instrument mobility, and tremor amplification, which may compromise surgical precision. Robot-assisted minimally invasive gastrectomy (RAMIG) addresses these challenges through high-definition three-dimensional imaging, tremor filtration, and enhanced instrument articulation, thereby improving precision, reducing intraoperative trauma and surgeon fatigue, and facilitating a shorter learning curve [7–10].

Beyond comparable oncological safety, existing evidence suggests that RAMIG may confer perioperative benefits such as reduced blood loss, shorter hospitalization, and potentially lower complication rates, albeit with longer operative duration [11–13]. However, its impact on postoperative pain management and opioid consumption remains inadequately characterized, with only limited retrospective data available [14].

Postoperative pain management is a critical determinant of surgical recovery, yet comparative evidence regarding analgesic requirements between robotic and open gastrectomy remains limited. The clinical significance extends well beyond patient comfort, as inadequate analgesia can delay gastrointestinal recovery and hospital discharge, while excessive opioid use carries risks including respiratory depression, delayed bowel function, and dependency, making opioid stewardship a central priority in perioperative care [15, 16].

Robotic surgery, with its enhanced dexterity, stable visualization, and reduced tissue manipulation, theoretically offers advantages that may translate into clinically meaningful reductions in postoperative pain and opioid consumption. However, standardized comparative data evaluating these outcomes under uniform multimodal analgesic protocols are scarce [14]. Such evidence is essential to inform surgical decision-making and optimize perioperative care pathways in gastric cancer treatment.

This study aimed to evaluate postoperative pain and opioid consumption between RAMIG and OG under a standardized multimodal analgesic protocol. Primary endpoints were cumulative morphine milligram equivalents (MME) and pain intensity, assessed by the numerical rating scale (NRS). Secondary endpoints included complication rates, intraoperative blood loss, postoperative intensive care/intermediate care (ICU/IMC) stay, total hospital length of stay, and surgical duration. We hypothesized that RAMIG would be associated with lower pain scores, reduced opioid requirements, and improved recovery trajectories, thereby addressing the clinically meaningful goals of opioid stewardship, complication prevention, and shorter hospitalization in gastric cancer care.

These findings could inform surgical decision-making and support a more patient-centered approach in gastric cancer surgery. Additionally, reduced ICU/IMC and hospital stay may translate into lower healthcare costs, which warrants further prospective cost-effectiveness analyses.

This retrospective cohort study has been reported in line with the STROBE [17] guidelines for observational studies and structured according to the STROCCS 2025 recommendations for surgical research [18].

## Material and methods

### Study design

This study presents a retrospective, single-center cohort study comparing the efficacy of RAMIG versus OG in 138 patients with resectable gastric cancer, performed at a tertiary care university hospital (University Hospital Muenster, Germany). The primary objective was to evaluate RAMIG's performance compared to OG in patients with resectable gastric cancer.

This retrospective study was approved by the Ethics Committee of the Medical Association of Westphalia-Lippe and the University of Muenster (Muenster, Germany) on March 11, 2022 (file number 2022-123-f-S) and was retrospectively registered in the German Clinical Trials Register (DRKS) under the identification number DRKS00036368. The study adhered to ethical standards outlined in the Declaration of Helsinki and Good Clinical Practice principles [19, 20].

Between May 2012 and August 2023, patients diagnosed with gastric cancer underwent either RAMIG utilizing the Da Vinci Surgery System (Intuitive Surgical Inc., Sunnyvale, CA) or conventional OG. The non-randomized, retrospective design may have introduced selection bias, as the surgical approach was determined by surgeon preference and institutional protocols.

Data were retrospectively extracted from electronic medical records. Due to the retrospective design, the requirement for informed consent was waived by the ethics committee. Written consent for scientific use of anonymized medical data was routinely obtained at hospital admission. Postoperative outcomes were assessed during the inpatient stay and until hospital discharge. No deviations from the initial study design or timeline occurred.

MME were calculated utilizing conversion tables provided by the Centers for Disease Control and Prevention [21] and the German Pain Society [22], based on the most recent versions available as of December 2022. These MME Conversion Factors were employed to standardize opioid dosages across various substances, ensuring accurate comparative analyses.

### Patients

Between May 2012 and August 2023, patients diagnosed with gastric cancer were identified through the hospital’s tumor registry and surgical databases. OG procedures were performed from May 2012 to June 2023, while RAMIG procedures were performed from September 2018 to August 2023.

Eligibility required age ≥ 18 years and resectable gastric cancer or adenocarcinoma of the esophagogastric junction, Siewert type II or III. Siewert type I tumors were excluded, as they are classified as distal esophageal cancers requiring Ivor–Lewis esophagectomy [23, 24]. Patients with peritoneal carcinomatosis, laparoscopic procedures, palliative indications, distant metastases, or hyperthermic intraperitoneal chemotherapy were excluded due to non-comparability of treatment protocols. All patients were required to receive epidural analgesia via epidural catheter for five postoperative days.

**Fig. 1:**
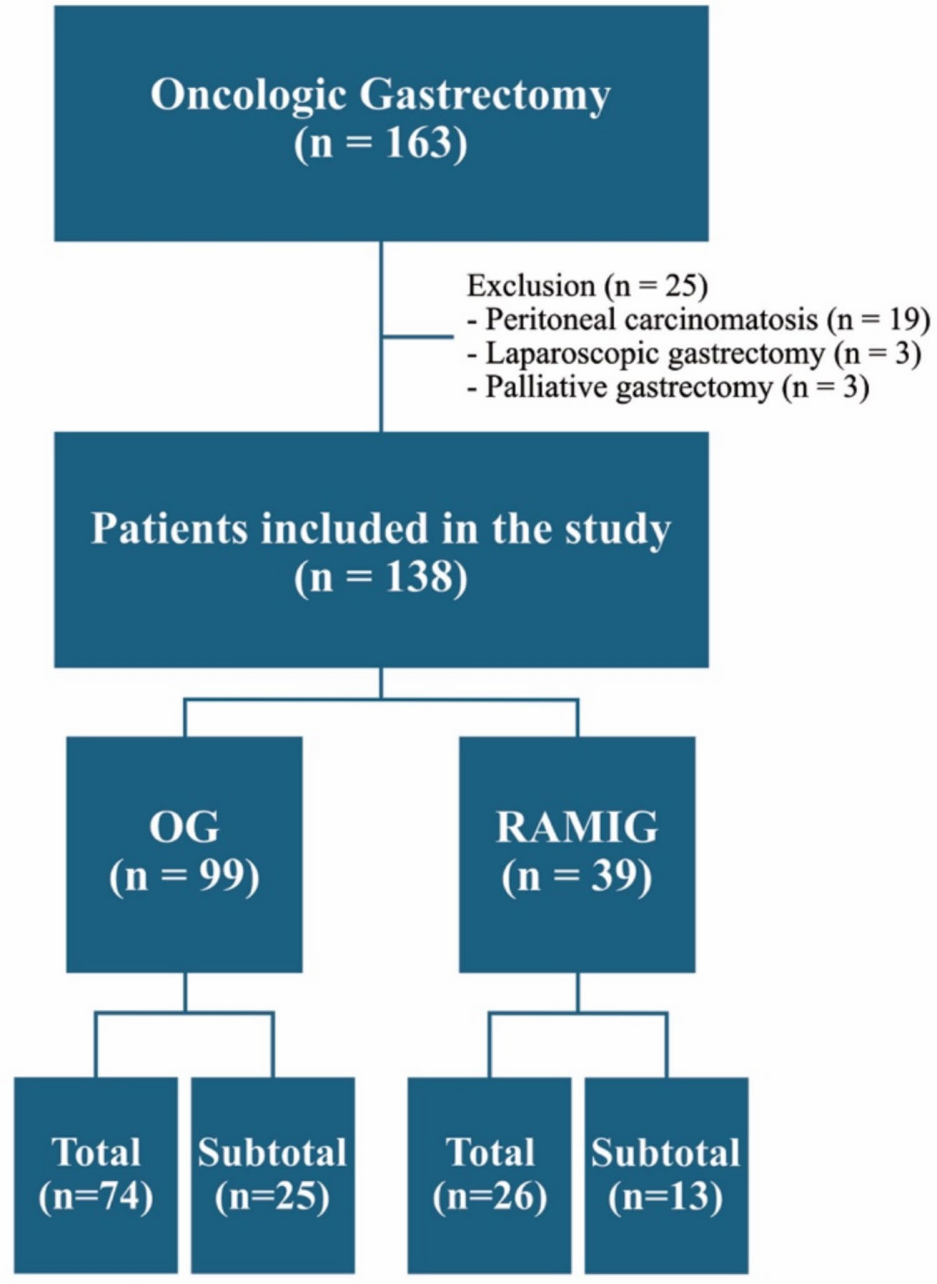
Flow chart of patient selection with exclusion of peritoneal carcinomatosis, laparoscopic and palliative gastrectomy, resulting in final allocation to OG and RAMIG groups stratified by resection extent (total vs. subtotal gastrectomy)

The study included 138 patients, with 99 undergoing OG (74 total gastrectomy, 25 subtotal gastrectomy) and 39 receiving RAMIG (26 total gastrectomy, 13 subtotal gastrectomy). See Fig. 1 for patient selection.

No formal power calculation was performed due to the retrospective design. The sample size included all consecutive eligible patients and was considered sufficient for meaningful statistical comparisons. No patient incentives were provided.

Preoperative diagnostics included ultrasound, esophagogastroduodenoscopy, endoscopic ultrasound, and thoracoabdominal CT. Diagnostic laparoscopy was performed in T3/T4 tumors to exclude peritoneal carcinomatosis. Treatment strategies were based on preoperative staging and tumor board recommendations, in accordance with German Cancer Society guidelines, and included neoadjuvant chemotherapy (FLOT or ECX/ECF/EOX), chemoradiotherapy, or primary surgery.

Perioperative management included epidural catheterization and prophylactic antibiotics (cefuroxime 3 g, metronidazole 500 mg) given 30 min preoperatively.

### Surgical technique

Both total and subtotal gastrectomies were included. A subgroup analysis was performed, comparing outcomes between OG and RAMIG separately within subtotal and total gastrectomies; results are provided in Supplementary Table S2. All procedures were performed with standardized D2 lymphadenectomy and Roux-Y reconstruction. Reconstruction techniques were procedure-specific: in total gastrectomy, esophagojejunostomy was performed using end-to-side anastomosis with a 25-mm circular stapler in both OG and RAMIG groups, while subtotal procedures employed side-to-side gastrojejunostomy with a 60-mm linear stapler in both groups. RAMIG was performed by surgeons with established expertise in robotic upper gastrointestinal surgery. All robotic gastrectomies were performed utilizing the Da Vinci XI System under a standardized protocol to ensure precision and reproducibility. The specimen was extracted via a standardized mini laparotomy in the left lumbar region using an Alexis wound retractor. The procedure commenced with greater omental mobilization and bursal exposure. The right gastroepiploic vessels were ligated proximally, followed by fundal dissection from pancreatic adhesions. Duodenal transection was performed distally to the pylorus using a 60-mm blue cartridge robotic stapler, followed by crural dissection for esophageal mobilization. D2 lymphadenectomy included transection of left gastric, right gastroepiploic, and right gastric vessels using a vessel sealer and Grena clips.

In total gastrectomies, esophageal transection occurred 2 cm above the gastroesophageal junction. A jejunal loop (40 cm from Treitz) was retrocolically positioned, followed by end-to-side esophagojejunostomy using a 25-mm circular stapler. In subtotal gastrectomies, gastric transection was performed, maintaining a gastric remnant, followed by side-to-side gastrojejunostomy using a 60-mm linear stapler. Jejunojejunostomy was performed 50 cm distal to the esophagojejunostomy using an Echelon Flex 60-mm blue cartridge stapler with reinforcing cross-stitches. Anastomotic integrity was confirmed by intraoperative leak testing. Foley and Robinson drains were placed at the duodenal stump and esophagojejunostomy, respectively. Finally, nasogastric tube placement was performed.

### Postoperative care

Clinical teams determined postoperative monitoring based on hemodynamic stability parameters. Patients were transferred to ICU or IMC units according to standardized criteria, while hemodynamically stable patients at low complication risk were transferred directly to the general surgical ward.

Pain management followed a multimodal protocol including epidural analgesia, NSAIDs (primarily metamizole), and patient-individualized opioid therapy administered via intravenous and oral routes according to clinical assessment and WHO analgesic ladder principles [25]. Although opioid therapy was individualized, all doses were standardized as MME to ensure comparability. Ondansetron was available as rescue antiemetic therapy. Epidural catheters were placed preoperatively, monitored by the acute pain team, and routinely removed by postoperative day five. Adjustments were made in collaboration with surgeons [26, 27]. Opioid administration was strictly inpatient-based, individualized, and systematically tapered before discharge according to institutional guidelines.

Nutritional support was initiated within 24 h postoperatively according to evidence-based guidelines. The protocol specified enteral feeding or restricted oral intake (≤ 500 mL/day) for three days, with gradual diet advancement from postoperative day five. Postoperative care included immediate mobilization with standardized physiotherapy, routine prophylactic drain placement [28], and nasogastric tubes were not routinely maintained postoperatively. Daily laboratory monitoring was performed for early complication detection, and in-house surgical endoscopy was readily available when clinically indicated.

Discharge criteria included stable vital signs, adequate oral intake, and absence of major complications. No deviations from standardized postoperative treatment protocols occurred during the study period.

### Endpoints

Baseline characteristics, including age, sex, body mass index (BMI), Charlson Comorbidity Index (CCI), American Society of Anesthesiologists (ASA) score, tumor type, preoperative tumor stage, and preoperative lymph node stage, were evaluated for comparability across study groups. This standardization ensures that observed outcome differences can be attributed to surgical technique rather than baseline demographic variations.

Primary endpoints comprised overall postoperative opioid consumption until discharge, expressed in cumulative MME, as well as weight-normalized MME and hospitalization-normalized MME. Given the standardized pain-adapted analgesic regimen across all patients, opioid consumption served as a validated primary endpoint for evaluating differential pain experiences between surgical approaches.

Pain intensity was assessed using NRS, ranging from 0 to 10, on postoperative days 1, 3, 5, and 7, differentiating between pain at rest and activity-related pain. For multiple daily assessments, mean pain scores were calculated. For statistical analysis, NRS scores were categorized into three groups: 0–3 (group 1), > 3–6 (group 2), and > 6–10 (group 3).

Secondary endpoints included length of postoperative ICU/IMC stay, total hospital stay, intraoperative blood loss, postoperative complications, and surgical duration. The assessment of complications encompassed pneumonia, anastomotic leakage, reoperation, pancreatic fistula formation, hospital mortality, and the proportion of patients experiencing severe postoperative complications (Clavien-Dindo ≥ 3b).

NRS data and time to first defecation were not fully available for all patients due to minor gaps in electronic medical record documentation.

### Statistical analysis

Statistical analysis was performed using SPSS version 29.0 (IBM Corp., Armonk, NY, USA). Nominal and ordinal variables were presented as frequencies and percentages. CCI was reported as an unaggregated median with interquartile range (IQR) and additionally grouped into categorical bands (1–2, 3–4, ≥ 5), with scores 6–21 aggregated as ≥ 5. NRS pain scores were averaged per postoperative day and, like other continuous variables, were reported as medians with IQR.

Statistical significance of observed differences was determined using Fisher’s exact test for nominal variables, with two-sided *p*-values. The Mann–Whitney *U* test was applied to all ordinal and metric variables. Statistical significance was defined as *p* < 0.05, with unadjusted *p*-values reported.

The bootstrap confidence intervals were calculated using the bias-corrected and accelerated method with 2000 samples.

## Results

### Patient characteristics

As detailed in Fig. 1, 163 patients were initially screened, with 25 excluded according to pre-defined criteria, resulting in 138 patients (99 OG, 39 RAMIG) included in the final analysis. Two robotic cases required conversion to open surgery. In accordance with the intention-to-treat principle, these patients were analyzed in the RAMIG group. Baseline characteristics, including demographics, comorbidities, and preoperative medication, were largely comparable between groups. Height was significantly higher in the RAMIG group (*p* = 0.023), while all other variables showed no statistically significant differences (all *p* > 0.05). Tumor type, location, and neoadjuvant treatment were similarly distributed. Full characteristics are shown in Table 1. See Supplementary Table S1 for full comorbidity breakdown.

**Table 1:** Patient demographics, comorbidity, and tumor characteristics

	OG (*n* = 99)	RAMIG (*n* = 39)	*p* value
Patient demographics			
Age (years)^c^	66 (55, 74)	64 (52, 71)	0.371
Age (years) grouped^a^			0.895
< 50^a^	14 (14.1)	7 (17.9)	
50-59^a^	18 (18.2)	6 (15.4)	
60-69^a^	30 (30.3)	13 (33.3)	
70-79^a^	24 (24.2)	10 (25.6)	
> 80^a^	13 (13.1)	3 (7.7)	
Sex (M:F)^a^	62 (62.6): 37 (37.4)	27 (69.2): 12 (30.8)	0.555
Female^a^	37 (37.4)	12 (30.8)	
Male^a^	62 (62.6)	27 (69.2)	
Height (cm)^c^	171 (164, 178)	176 (168, 182)	0.023^*^
Weight (kg)^c^	78 (65, 85)	75 (65, 92)	0.728
BMI (kg/m^2^)^c^	25.34 (22.43, 29.07)	25.24 (21.64, 28,08)	0.503
BMI (kg/m^2^) grouped^a^			0.905
< 20^a^	9 (9.1)	4 (10.3)	
20-30^a^	72 (72.7)	29 (74.4)	
> 30^a^	18 (18.2)	6 (15.4)	
Charlson Comorbidity Index^c^	5 (4, 6)	4 (4, 6)	0.319
Charlson Comorbidity Index Bands			
1–2^a^	13 (13.1)	5 (12.8)	
3–4^a^	27 (27.3)	15 (38.5)	
≥ 5^a^	59 (59.6)	19 (48.7)	
ASA Score^b^			0.086
1^b^	2 (2.0)	1 (2.6)	
2^b^	48 (48.5)	25 (64.1)	
3^b^	48 (48.5)	13 (33.3)	
4^b^	1 (1.0)	0 (0)	
Tumor characteristics			
Tumor entity^a^			0.809
Adenocarcinoma^a^	93 (94.9)	37 (94.9)	
Sarcoma^a^	2 (2.0)	0 (0.0)	
GIST^a^	3 (3.1)	2 (5.1)	
Tumor localization^a^			0.740
Cardia^a^	24 (24.2)	6 (15.4)	
Fundus^a^	3 (3.0)	1 (2.6)	
Corpus^a^	41 (41.4)	16 (41.0)	
Antrum^a^	29 (29.3)	15 (38.5)	
Diffuse^a^	2 (2.0)	1 (2.6)	
Neoadjuvant therapy^a^	50 (50.5)	25 (64.1)	0.185
Chemotherapy^a^	49 (98.0)	23 (92.0)	
Chemoradiotherapy^a^	1 (2.0)	2 (8.0)	
Lauren classification^a^			0.201
Intestinal type^a^	46 (47.9)	13 (33.3)	
Diffuse type^a^	44 (45.8)	20 (51.3)	
Mixed type^a^	3 (3.1)	3 (7.7)	
Pathological nodal status^b^			0.351
0^b^	43 (43.9)	20 (52.6)	
1^b^	18 (18.4)	6 (15.8)	
2^b^	12 (12.2)	3 (7.9)	
3^b^	18 (18.4)	9 (23.7)	
4^b^	7 (7.1)	0 (0.0)	
Positive lymph nodes^c^	1.0 (0.0, 7.25)	0.0 (0.0, 6.25)	0.364
Harvested lymph nodes^c^	25.5 (18.0, 34.25)	29.0 (21.0, 34.25)	0.139
UICC stage grouped ^a^			0.337
0-II^a^	56 (56.6)	26 (66.7)	
III-IV^a^	43 (43.4)	13 (33.3)	

*M* male, *F* female, *BMI* body mass index, *ASA* American Society of Anesthesiologists Physical Status Classification System, *GIST* gastrointestinal stromal tumor, *UICC* Union for International Cancer Control, *MWU* Mann–Whitney U, *IQR* interquartile range

^a^Data are presented as absolute numbers and percentages; statistical comparison performed using Fisher’s exact test (nominal variables)

^b^Data are presented as absolute numbers and percentages; statistical comparison performed using the MWU test (ordinal variables)

^c^Data are presented as median and interquartile range (IQR); statistical comparison performed using the MWU test (continuous variables)

^*^p < 0.05 was considered statistically significant

### Primary outcomes: opioid consumption and pain scores

RAMIG patients demonstrated significantly lower postoperative opioid requirements compared to OG patients across multiple analyses. The total postoperative MME was substantially reduced in the RAMIG group (median 162.0 mg MME, IQR 90.0–255.0) compared to the OG group (median 240.0 mg, IQR 130.0–570.0; *p* = 0.002).

**Fig. 2:**
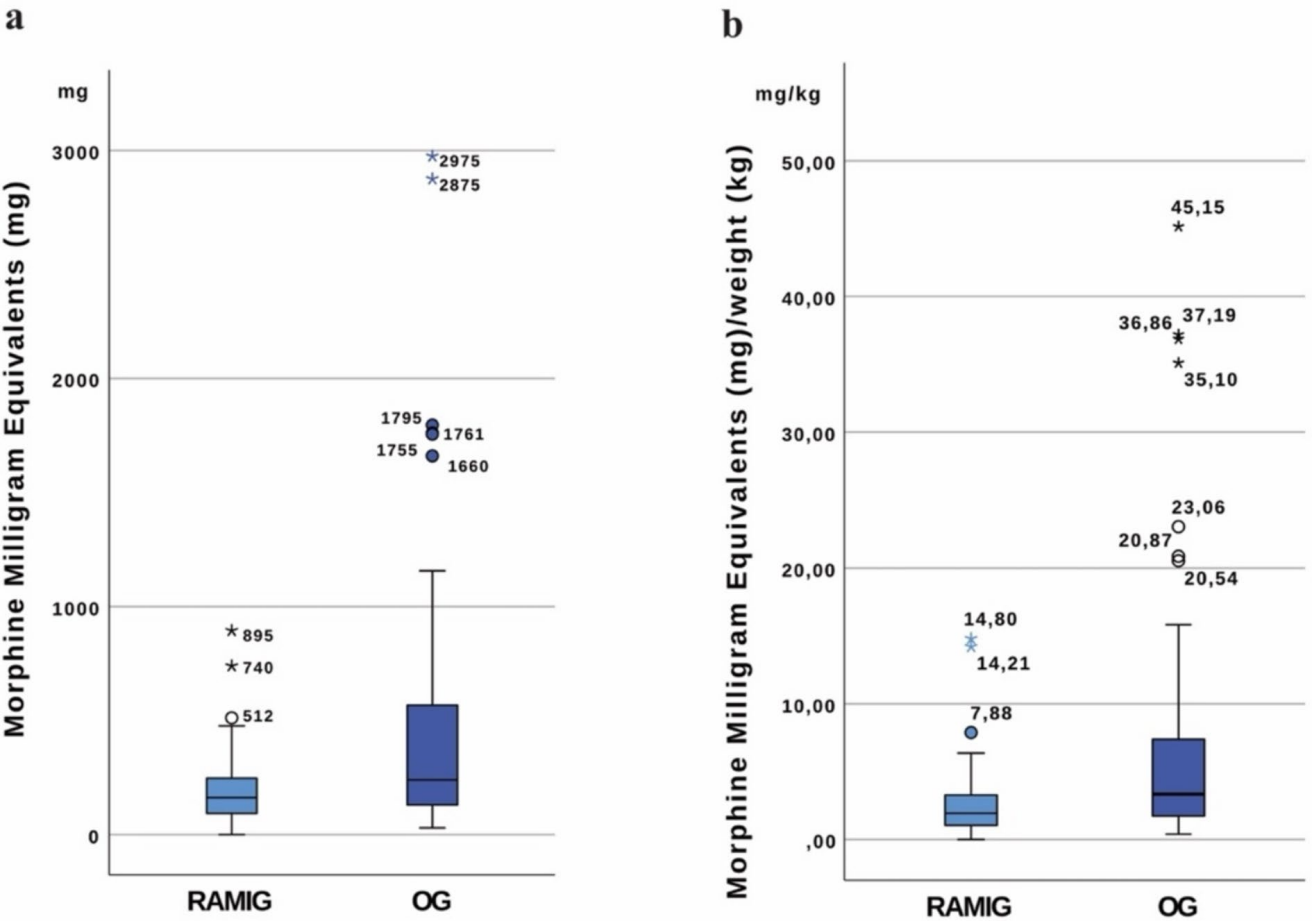
Box-plot chart illustrating opioid consumption patterns in RAMIG and OG groups: **a** total MME and **b** MME per kilogram body weight; boxes represent interquartile range, whiskers extend to the most extreme values within 1.5 × IQR, circles denote mild outliers, and asterisks denote extreme outliers

**Fig. 3:**
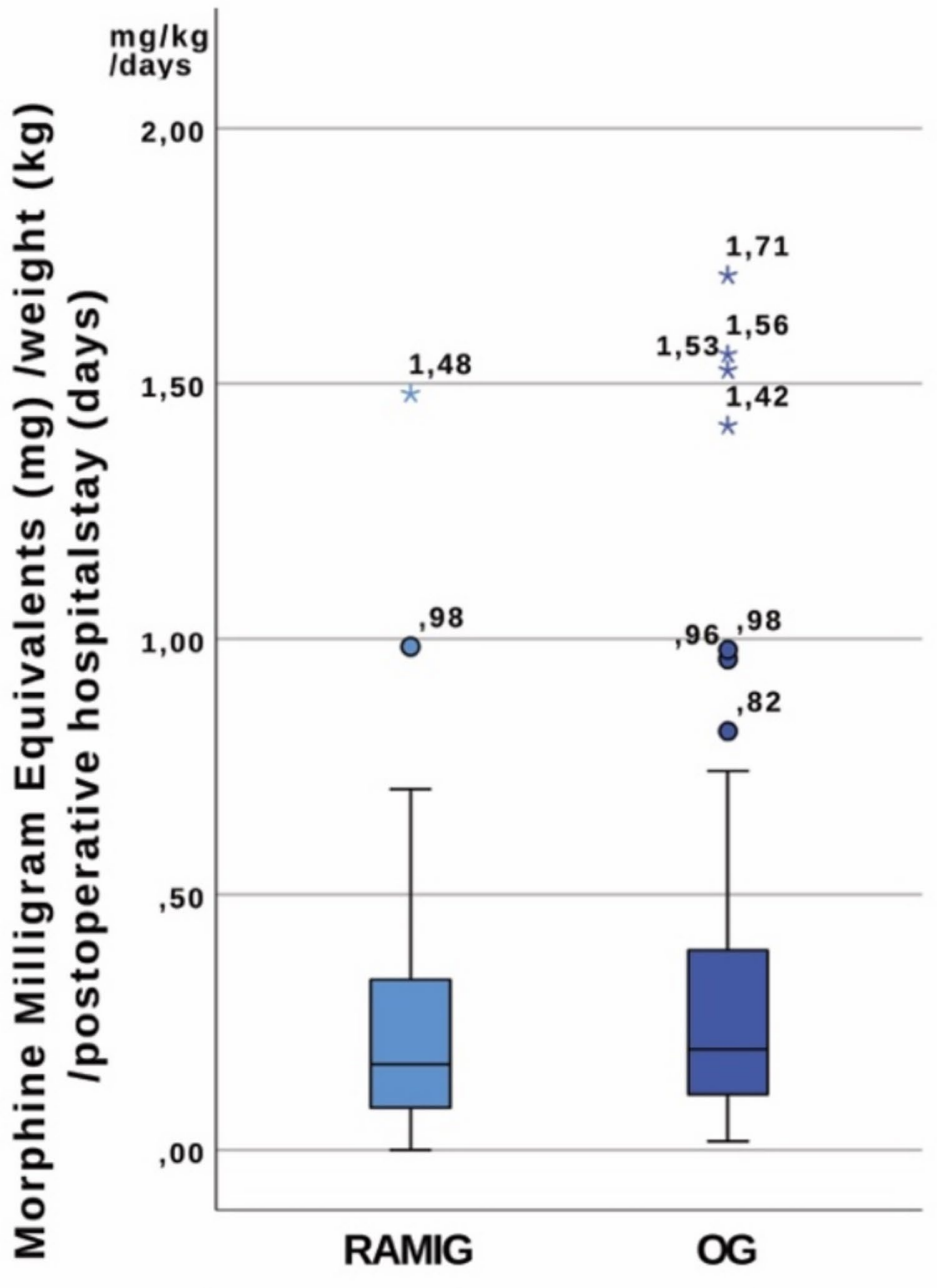
Box-plot chart illustrating opioid consumption patterns in RAMIG and OG groups (MME per kilogram body weight per day; boxes represent interquartile range, whiskers extend to the most extreme values within 1.5 × IQR, circles denote mild outliers, and asterisks denote extreme outliers

When adjusted for patient weight, the difference became even more pronounced, with RAMIG patients requiring less MME per kilogram body weight (median 1.92 mg/kg, IQR 0.98–3.33) than OG patients (median 3.33 mg/kg, IQR 1.69–7.5; *p* < 0.001). Analysis of daily weight-adjusted consumption showed a similar trend toward reduced opioid use in RAMIG patients (median 0.17 mg/kg/day, IQR 0.08–0.33) compared to OG patients (median 0.20 mg/kg/day, IQR 0.11–0.40), though this difference did not reach statistical significance (*p* = 0.322). Figures 2 and 3 present box-plot charts illustrating these opioid consumption patterns. Five patients (12.8%) in the RAMIG group remained opioid-free, whereas none in the OG group did (*p* = 0.001).

**Fig. 4:**
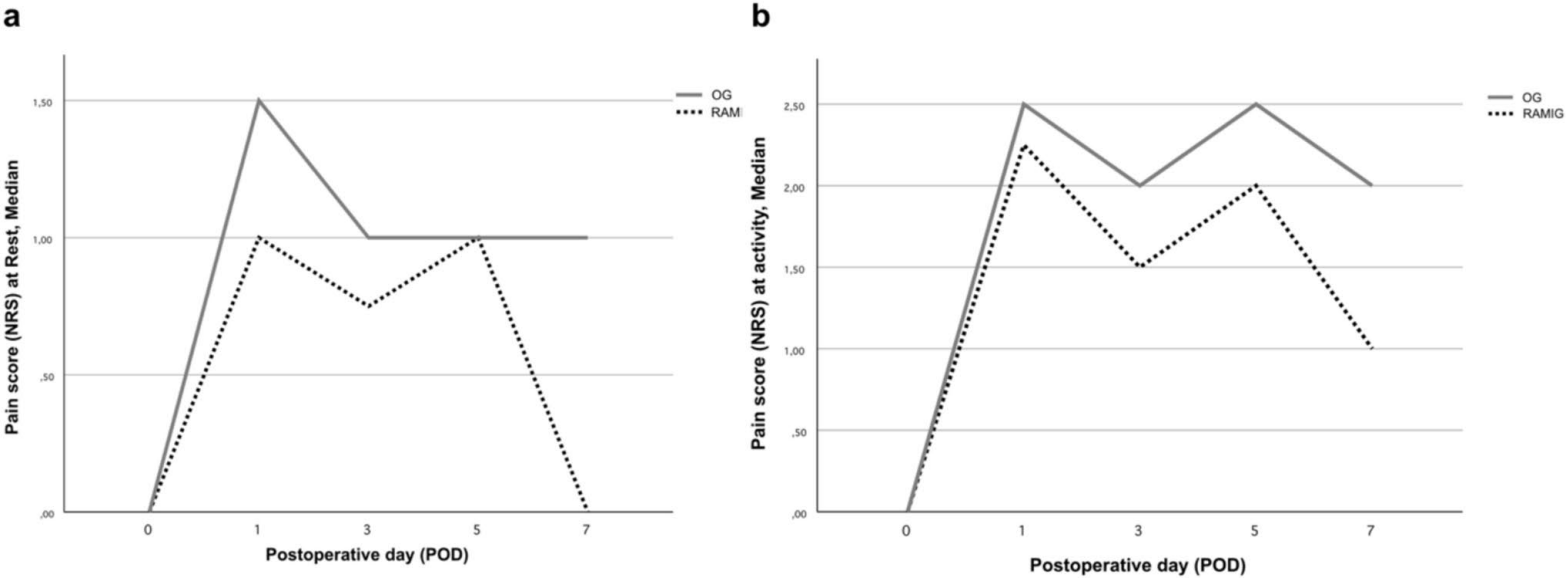
Line charts illustrating postoperative pain trajectories (NRS scores) in RAMIG and OG groups: **a** pain at rest, **b** pain during activity. Lines indicate median values per postoperative day

On postoperative day 5, the RAMIG group exhibited significantly lower activity-related pain levels compared with the OG group (*p* = 0.011). By postoperative day 7, both activity-related and resting pain levels were significantly lower in the RAMIG group (activity: *p* = 0.002; rest: *p* = 0.005). Although the RAMIG group demonstrated reduced pain scores at other measurement timepoints, these differences did not reach statistical significance. Postoperative pain trajectories are depicted in Fig. 4, showing median NRS scores per postoperative day for RAMIG and OG patients, separately for (a) rest and (b) activity.

### Secondary outcomes: perioperative parameters

The operative duration was significantly prolonged in the RAMIG group (median 7:12 h, IQR 6:04–8:24) compared to the OG group (median 4:49 h, IQR 4:02–5:43; *p* < 0.001). Intraoperative blood loss was markedly lower in the RAMIG group (median 100 mL, IQR 50–500) compared with the OG group (median 300 mL, IQR 100–550; *p* = 0.009). All patients received standardized perioperative antibiotic prophylaxis.

RAMIG patients demonstrated significantly shortened ICU/IMC stays compared to OG patients (*p* < 0.001). Similarly, total hospitalization duration was reduced in the RAMIG group (*p* < 0.001). Bootstrap 95% confidence interval for mean ICU/IMC stay reduction in the RAMIG group relative to the OG group ranged from 3.69 to 9.68 days, with a mean difference of 6.23 days. Regarding total postoperative stay reduction, the bootstrap 95% confidence interval spanned 4.48 to 12.15 days, with a mean difference of 8.46 days. Time to first defecation demonstrated no significant difference between the groups (*p* = 0.821).

### Subgroup analysis

Subgroup analysis revealed differential treatment effects between gastrectomy types (Supplementary Table S2). In total gastrectomies, RAMIG demonstrated more pronounced advantages including significantly reduced opioid consumption (268.5 vs 188 mg MME, *p* = 0.013), lower blood loss (375 vs 75 mL, *p* = 0.008), and higher rates of opioid-free recovery (0 vs 4 patients, *p* = 0.004). In contrast, subtotal gastrectomies showed smaller, non-significant differences in opioid consumption (200 vs 110 mg MME, *p* = 0.064) and blood loss (200 vs 200 mL, *p* = 0.649).

### Postoperative complications

Severe complications (Clavien-Dindo ≥ 3b) occurred in 12.8% of the RAMIG group versus 19.2% of the OG group, without statistical significance (*p* = 0.460).

Neither pneumonia incidence (*p* = 0.468) nor anastomotic leakage rates (*p* = 0.779) differed significantly between groups. Pancreatic fistulas occurred exclusively in the OG group (three cases), though this difference was not statistically significant (*p* = 0.559).

The RAMIG group exhibited lower reoperation rates (2.6% versus 14.1%; *p* = 0.067). Reoperative indications encompassed anastomotic leakage, fascial dehiscence, hepatic capsule hematoma, laparotomy wound infection, partial colonic resection for hypoperfusion, continuity restoration following emergency surgery, and lavage for early pancreatitis.

Mortality rates were 0.0% in the RAMIG group and 2.0% in the OG group (*p* = 1.0).

### Key results

In summary, RAMIG demonstrated statistically significant advantages, including lower median opioid consumption (162 vs 240 mg MME, *p* = 0.002), shorter mean ICU/IMC stays (6.2-day reduction, *p* < 0.001), and reduced median intraoperative blood loss (100 vs 300 mL, *p* = 0.009).

Comprehensive postoperative outcomes are presented in Table 2.

**Table 2:** Perioperative parameters and clinical outcomes

	OG (*n* = 99)	RAMIG (*n* = 39)	*p*-value
Operative data			
Type of gastrectomy^a^			0.398
Subtotal^a^	25 (25.3)	13 (33.3)	
Total^a^	74 (74.7)	26 (66.7)	
Blood loss (mL)^c^	300 (100, 550)	100 (50, 500)	0.009^*^
Surgery duration (hh:mm)^c^	4:49 (4:02, 5:43)	7:12 (6:04, 8:24)	< 0.001^*^
Perioperative antibiotic use^a^	99 (100)	39 (100)	Perioperative antibiotics is a constant
Conversion^a^		2 (5.1)	
Analgesic use			
Total MME (mg)^c^	240.0 (130.0, 570.0)	162.0 (90.0, 255.0)	0.002^*^
MME per kg body weight (mg/kg)^c^	3.33 (1.69, 7.5)	1.92 (0.98, 3.33)	< 0.001^*^
MME per kg per POD (mg/kg/day)^c^	0.2 (0.11, 0.40)	0.17 (0.08, 0.33)	0.322
Opioid-free patients^a^	0 (0.0)	5 (12.8)	0.001^*^
Pain score (NRS)—rest			
NRS rest PRE^c^	0.0 (0.0, 0.0)	0.0 (0.0, 0.0)	0.364
NRS rest POD 1^c^	1.50 (1.0, 2.0)	1.0 (0.13, 2.5)	0.461
NRS rest POD 3^c^	1.0 (0.0, 2.0)	0.75 (0.0,2.0)	0.673
NRS rest POD 5^c^	1.0 (0.5, 2.3)	1.0 (0.0, 2.0)	0.112
NRS rest POD 7^c^	1.0 (0.0, 2.38)	0.0 (0.0, 1.38)	0.005^*^
Pain score (NRS)—activity			
NRS activity PRE^c^	0.0 (0.0, 0.0)	0.0 (0.0, 1.0)	0.245
NRS activity POD 1^c^	2.50 (2.0, 4.0)	2.25 (1.0, 4.0)	0.187
NRS activity POD 3^c^	2.0 (2.0, 3.0)	1.5 (0.5, 3.0)	0.090
NRS activity POD 5^c^	2.50 (2.0, 4.0)	2.0 (1.0 2.5)	0.011^*^
NRS activity POD 7^c^	2.0 (0.5, 3.0)	1.0 (0.0, 2.0)	0.002^*^
Grouped pain scores (NRS)			
NRS rest POD 1 grouped^c^	1.0 (1.0, 1.0)	1.0 (0.25, 1.0)	0.093
NRS rest POD 3 grouped^c^	1.0 (0.0, 1.0)	1.0 (0.0, 1.0)	0.158
NRS rest POD 5 grouped^c^	1.0 (1.0, 1.0)	1.0 (0.0, 1.0)	0.511
NRS rest POD 7 grouped^c^	1.0 (0.0, 1.0)	0.0 (0.0, 1.0)	0.006^*^
NRS activity POD 1 grouped^c^	1.0 (1.0, 2.0)	1.0 (1.0, 2.0)	0.358
NRS activity POD 3 grouped^c^	1.0 (1.0, 1.0)	1.0 (1.0, 1.0)	0.186
NRS activity POD 5 grouped^c^	1.0 (1.0, 2.0)	1.0 (1.0, 1.0)	0.045^*^
NRS activity POD 7 grouped^c^	1.0 (1.0, 1.0)	1.0 (0.0, 1.0)	0.019^*^
Postoperative outcomes			
ICU/IMC length of stay (days)^c^	5 (2, 7)	1 (1, 2)	< 0.001^*^
Postoperative hospital stay (days)^c^	17 (12, 24)	9 (7, 13)	< 0.001^*^
Time to first defecation (days)^c^	3 (3, 4)	3 (2, 3)	0.821
Postoperative complications			
Complications (MCDC)^a^			
Grade 0^a^	3 (3.0)	10 (25.6)	
Grade 1^a^	9 (9.1)	6 (15.4)	
Grade 2^a^	27 (27.3)	12 (30.8)	
Grade 3a^a^	41 (41.4)	6 (15.4)	
Grade 3b^a^	9 (9.1)	3 (7.7)	
Grade 4a^a^	6 (6.1)	2 (5.1)	
Grade 4b^a^	3 (3.0)	0 (0.0)	
Grade 5^a^	1 (1.0)	0 (0.0)	
Severe complications (MCDC ≥ 3b)^a^	19 (19.2)	5 (12.8)	0.460
Pneumonia^a^	6 (6.1)	4 (10.3)	0.468
Anastomotic leakage^a^	13 (13.1)	4 (10.3)	0.779
Pancreatic fistula^a^	3 (3.0)	0 (0.0)	0.559
Reoperation^a^	14 (14.1)	1 (2.6)	0.067
Hospital mortality^a^	2 (2)	0 (0)	1.000
Hospital mortality^a^	2 (2)	0 (0)	1.000

*ICU/IMC* intensive care unit/intermediate care, *MCDC* modified Clavien–Dindo classification, *MME* morphine milligram equivalent, *POD* postoperative day, *NRS* numerical rating scale, *PRE* preoperative, *MWU* Mann–Whitney U, *IQR* interquartile range

^a^Data are presented as absolute numbers and percentages; statistical comparison performed using Fisher’s exact test (nominal variables)

^c^Data are presented as median and interquartile range (IQR); statistical comparison performed using the MWU test (continuous variables)

^*^p < 0.05 was considered statistically significant

## Discussion

### Principal findings

This retrospective cohort study demonstrated potential advantages of RAMIG over conventional OG, primarily regarding postoperative pain and opioid consumption. RAMIG patients required significantly less opioid medication overall, although this difference diminished when normalized for hospital stay, likely reflecting the shorter hospitalization period in the RAMIG group.

Pain intensity was generally low in both groups under multimodal analgesia, but significantly lower NRS scores were observed in the RAMIG group on postoperative days 5 and 7, particularly during mobilization. These findings suggest that RAMIG may facilitate enhanced recovery through improved pain control and reduced analgesic requirements.

We hypothesize that the removal of the epidural catheter on postoperative day 5 may reveal the impact of surgical techniques on pain levels, with RAMIG-associated reductions in surgical trauma likely contributing to these differences [11]. These results underscore the potential pain-reducing benefits of RAMIG during the early recovery phase.

In the RAMIG group, reduced opioid usage was observed alongside lower pain intensity. Although such reductions may theoretically translate into fewer opioid-related complications, including bowel dysfunction, nausea, and respiratory depression, our retrospective design did not allow for systematic assessment of these specific adverse events. Future prospective studies are needed to confirm whether decreased opioid use after RAMIG indeed leads to clinically meaningful reductions in opioid-related morbidity [16].

Our findings support previous research showing reduced opioid use in RAMIG compared to OG. However, a prior study found no significant difference in pain scores despite lower opioid use in the robotic group [14]. In contrast, our study identified significant pain reductions on specific postoperative days, suggesting an early pain relief advantage. Variations in pain assessment, analgesia protocols, or sample sizes may explain these differences. Nonetheless, both studies consistently demonstrate that RAMIG is associated with reduced opioid consumption.

Although our analysis focused on robotic versus open gastrectomy, comparison with laparoscopic approaches remains clinically relevant as laparoscopy is the established minimally invasive standard. Recent meta-analyses and reviews confirm comparable oncological safety between robotic and laparoscopic gastrectomy, while reporting perioperative advantages of the robotic approach such as reduced blood loss and shorter hospital stay [5, 13]. However, none have systematically evaluated postoperative pain or opioid consumption. Limited evidence from bariatric cohorts suggests modest reductions in pain and opioid requirements with robotic surgery [29] but dedicated data in oncological gastrectomy are lacking. Our study therefore contributes to addressing this gap by directly analyzing analgesic requirements in gastric cancer surgery.

Effective pain management, characterized by low pain levels and reduced opioid use, is essential for minimizing chronic postoperative pain risk [30–32] and promoting early mobilization, faster recovery, and improved quality of life [31, 33–35]. Balancing opioid side effects against their benefits for patient comfort remains crucial.

Beyond pain management, RAMIG was associated with a shorter ICU/IMC stay (*p* < 0.001) and hospital stay (*p* < 0.001). ICU/IMC stay was reduced on average by 6.23 days. Total hospital stay was reduced on average by 8.46 days. Both differences were statistically significant. Intraoperative blood loss was significantly lower in RAMIG (*p* = 0.009), potentially reducing transfusion requirements and related complications [5, 11, 12, 36]. Despite longer operative times (*p* < 0.001), this is expected to improve with increasing surgical experience and advancements in robotic technology [9, 10, 37, 38]. These results indicate an advantage in early postoperative recovery, likely due to the minimally invasive nature of RAMIG, which reduces surgical trauma, decreases pain perception, and facilitates earlier return to oral intake [11, 12, 37]. The shorter ICU/IMC stay in RAMIG may also be attributable to reduced blood loss and reduced surgical trauma, contributing to lower risks of hemodynamic instability and faster stabilization of vital parameters [6, 8, 39].

The longer ICU/IMC and hospital stays in our cohort (OG: median 5 and 17 days; RAMIG: 1 and 9 days) compared to randomized trials such as LOGICA (median 7–8 days) [6] and other series reporting 9.6 vs. 13.4 days [40] likely reflect institutional management strategies rather than the surgical approach. These include conservative ICU/IMC admission criteria, standardized perioperative care, and immediate access to in-house endoscopy for early complication detection. While this patient-centered practice may prolong hospital stay compared with accelerated discharge protocols, it ensures consistent safety and provides a robust framework for comparing surgical techniques. Our findings suggest that RAMIG could facilitate shorter stays in future practice.

Our findings align with existing literature, supporting RAMIG as a viable alternative to OG, with specific advantages such as reduced hospital stay (*p* < 0.001) and intraoperative blood loss (*p* = 0.009) [12, 39, 41].

Postoperative complication rates were comparable between groups, with no significant difference in severe complications (Clavien-Dindo ≥ 3b). RAMIG had lower rates of pancreatic fistulas (0% vs. 3%) and reoperations (2.6% vs. 14.1%), though these differences were not statistically significant. Evidence remains inconsistent, with some meta-analyses reporting significant differences in postoperative complication rates [12, 39] while others do not [37, 42]. In our cohort, severe complications (Clavien-Dindo ≥ 3b) were comparable, but RAMIG had a lower overall rate (12.8% vs. 19.2%).

Anastomotic leakage occurred in 10.3% of the RAMIG and 13.1% of OG patients (*p* = 0.779). These rates are somewhat higher than typically reported in randomized trials and meta-analyses [11–13], which may reflect our institution’s stricter postoperative surveillance, broader classification criteria, and inclusion of minor leaks not requiring reoperation, supported by longstanding in-house surgical endoscopy expertise. Consistent with recent network meta-analyses [11, 13], leakage rates did not differ significantly between robotic, laparoscopic, and open gastrectomy, highlighting that factors beyond surgical technique, such as anastomotic tension, vascular supply, and comorbidities, influence outcomes [11].

The elevated leakage rates in our cohort also raise questions about potential contributing factors. While direct clinical evidence linking postoperative opioid consumption to anastomotic leakage after gastrectomy is lacking, chronic preoperative opioid use has been associated with increased leakage risk in large colorectal cohorts [43]. Whether postoperative opioid exposure similarly contributes to impaired healing in gastric surgery remains uncertain and requires further investigation.

Overall, our safety findings are consistent with recent systematic reviews and meta-analyses confirming comparable safety between robotic and conventional approaches [8, 11, 13].

Beyond overall safety considerations, subgroup analysis indicated more pronounced advantages of RAMIG in total gastrectomies, which represented the majority of procedures, while subtotal procedures showed consistent but non-significant trends, likely reflecting smaller sample sizes. These findings warrant confirmation in larger prospective studies and may help refine patient selection for robotic approaches.

### Limitations

This single-center retrospective study is limited by selection bias and reduced generalizability. Confounders include temporal variation, surgeon differences despite standardized protocols, learning curves, and evolving surgical practice.

The retrospective design limited pain documentation and precluded analysis of opioid-related adverse effects. Time to first rescue opioid administration was not systematically documented. Cumulative MME was analyzed as a comprehensive measure of overall analgesic burden. Patient-level biases include expectation differences, placebo response, and variable responses to standardized pain management protocols.

Nevertheless, this study has several strengths, including standardized perioperative protocols, comprehensive pain assessment using validated NRS scores, systematic application of the Clavien–Dindo classification for complications, and inclusion of patient-centered outcomes such as pain and opioid consumption, which directly impact postoperative recovery and quality of life.

### Relevance and implications

The improved short-term outcomes in pain reduction, opioid consumption, and hospitalization duration support RAMIG as a patient-centered alternative to OG. These advantages likely result from the intrinsic technical benefits of robotic systems, such as enhanced precision, stability, and control, rather than surgeon experience alone, thereby contributing to the growing evidence base for RAMIG in gastric cancer surgery.

To enhance future research quality, prospective randomized controlled trials with larger sample sizes, standardized surgeon experience levels, and inclusion of long-term functional and oncological outcomes are recommended. Multi-center studies would improve generalizability and reduce single-institution bias.

## Conclusion

RAMIG demonstrates significant perioperative advantages over OG, including reduced postoperative pain, lower opioid requirements, decreased blood loss, and shortened ICU/IMC and hospital stays. Although associated with longer operative times, its minimally invasive nature facilitates accelerated recovery and represents a more patient-centered approach with demonstrated potential to improve short-term outcomes. Further randomized controlled trials and multicenter studies are warranted to validate these findings and define RAMIG’s role within standard surgical practice for gastric cancer.

## Supplementary Information

Below is the link to the electronic supplementary material.

Supplementary file1 (DOCX 29 KB)

## Data Availability

The data supporting the findings of this study are not publicly available due to privacy and ethical restrictions.
