# Supplementary material for: Enhanced recovery and reduced opioid requirements following robot-assisted minimally invasive gastrectomy: a retrospective cohort study
Source: J Robot Surg. 2025 Oct 2;19(1):653. doi: 10.1007/s11701-025-02839-8 (PMC12491087; doi:10.1007/s11701-025-02839-8)
Supplement: Supplementary file 1 — Supplementary file1 (DOCX 29 KB) [file 11701_2025_2839_MOESM1_ESM.docx]

**Supplementary Tables**

**Supplementary Table S1.:** Detailed distribution of comorbidities according to Charlson Comorbidity Index (CCI)

| Comorbidity Profile | OG (n = 99) | RAMIG (n = 39) | p-value |
| --- | --- | --- | --- |
| Diabetes mellitus^a^ | 16 (16.2) | 4 (10.3) | 0.435 |
| Diabetes with secondary complications^a^ | 6 (6.1) | 1 (2.6) | 0.673 |
| Cerebrovascular disease^a^ | 8 (8.1) | 5 (12.8) | 0.517 |
| Dementia^a^ | 0 (0.0) | 2 (5.1) | 0.078 |
| Myocardial infarction^a^ | 6 (6.1) | 0 (0.0) | 0.184 |
| Coronary artery disease^a^ | 13 (13.1) | 2 (5.1) | 0.232 |
| Congestive heart failure^a^ | 1 (1.0) | 2 (5.1) | 0.193 |
| Renal replacement therapy^a^ | 2 (2.0) | 0 (0.0) | 1.000 |
| Chronic pulmonary disease^a^ | 17 (17.2) | 6 (15.4) | 1.000 |
| COPD^a^ | 8 (8.1) | 1 (2.6) | 0.445 |
| Connective tissue disease^a^ | 4 (4.0) | 1 (2.6) | 1.000 |
| Peptic ulcer disease^a^ | 7 (7.1) | 6 (15.4) | 0.192 |
| Mild liver disease^a^ | 11 (11.1) | 1 (2.6) | 0.178 |
| Severe liver disease^a^ | 5 (5.1) | 0 (0.0) | 0.321 |
| Hemiplegia^a^ | 1 (1.0) | 0 (0.0) | 1.000 |
| Lymphoma^a^ | 2 (2.0) | 0 (0.0) | 1.000 |
| Metastatic solid tumor^a^ | 7 (7.1) | 2 (5.1) | 1.000 |
| Peripheral vascular disease^a^ | 5 (5.1) | 1 (2.6) | 1.000 |
| Antihypertensive medications^a^ | 54 (54.5) | 20 (51.3) | 0.851 |
| Corticosteroid therapy^a^ | 5 (5.1) | 2 (5.1) | 1.000 |
| Immunosuppressive therapy^a^ | 2 (2.0) | 2 (5.1) | 0.317 |
| Anticoagulation therapy^a^ | 21 (21.2) | 8 (20.5) | 1.000 |

^a^ Data are presented as absolute numbers and percentages; statistical comparison performed using Fisher’s exact test (nominal variables).
Abbreviations: COPD, Chronic obstructive pulmonary disease.

**Supplementary Table S2.:** Extent of resection (subtotal vs. total gastrectomy) and reconstruction techniques by surgical approach (OG vs. RAMIG)

| **TOTAL Procedure** | OG (n=74) | RAMIG (n=26) | p-value | |  |
| --- | --- | --- | --- | --- | --- |
| Postoperative hospital stay (days)^c^ | 17.5 (12, 24) | 9 (8.75, 15.25) | <0.001^*^ | |  |
| ICU/IMC length of stay (days)^c^ | 5 (2, 8) | 1 (1, 2.25) | <0.001^*^ | |  |
| Surgical duration (hh:mm) ^c^ | 4:56 (4:16, 5:53) | 7:27 (6:08, 8:49) | <0.001^*^ | |  |
| UICC grouped^a^ |  |  | 0.174 | |  |
| 0-II^a^ | 36 (48.6) | 17 (65.4) |  | |  |
| III-IV^a^ | 38 (51.4) | 9 (34.6) |  | |  |
| Pathological Nodal Status ^b^ | 1 (0, 3) | 0 (0, 2.25) | 0.087 | |  |
| Positive lymph nodes^c^ | 2 (0, 8) | 0 (0, 6.25) | 0.073 | |  |
| Harvested lymph nodes^c^ | 26.5 (20.75, 35.25) | 29 (21.75, 35.25) | 0.606 | |  |
| NRS rest PRE^c^ | 0 (0,0) | 0 (0,0) | 0.740 | |  |
| NRS rest POD 1^c^ | 1.5 (1, 2) | 1 (0, 2) | 0.310 | |  |
| NRS rest POD 3^c^ | 1 (0, 1.5) | 0.5 (0, 2) | 0.561 | |  |
| NRS rest POD 5^c^ | 1 (0.5, 2.08) | 1 (0, 2.5) | 0.631 | |  |
| NRS rest POD 7^c^ | 1 (0, 2.5) | 0 (0, 1) | 0.003^*^ | |  |
| NRS activity PRE^c^ | 0 (0,0) | 0 (0,1) | 0.236 | |  |
| NRS activity POD 1^c^ | 2.5 (2, 4) | 2 (1, 4) | 0.093 | |  |
| NRS activity POD 3^c^ | 2 (1.88, 4) | 2 (0, 3.5) | 0.247 | |  |
| NRS activity POD 5^c^ | 2.25 (0.88, 4.0) | 2 (1, 2.5) | 0.167 | |  |
| NRS activity POD 7^c^ | 2.25 (0.88, 4) | 1 (0, 1.88) | 0.002^*^ | |  |
| Total MME (mg)^c^ | 268.5 (133.75, 597.25) | 188 (101.25, 264) | 0.013^*^ | |  |
| MME per kg body weight (mg/kg)^c^ | 3.65 (1.75, 8.13) | 2.43 (1.35, 3.62) | 0.015^*^ | |  |
| MME per kg per POD (mg/kg/day)^c^ | 0.21 (0.10, 0.45) | 0.17 (0.06, 0.38) | 0.245 | |  |
| Blood loss (mL)^c^ | 375 (100, 612) | 75 (50, 425) | 0.008^*^ | |  |
| Opioid-free patients ^a^ | 0 (0.0) | 4 (15.4) | 0.004^*^ | |  |
| Severe complications  (MCDC ≥ 3b)^a^ | 16 (21.6) | 5 (19.2) | 1.0 | |  |
| Pneumonia^a^ | 5 (6.8) | 3 (11.5) | 0.425 | |  |
| Anastomotic leakage^a^ | 9 (12.2) | 4 (15.4) | 0.737 | |  |
| Reoperation^a^ | 11 (14.9) | 1 (3.8) | 0.177 | |  |
| Hospital mortality^a^ | 1 (1.4) | 0 (0.0) | 1.0 | |  |
| Pancreatic fistula^a^ | 3 (4.1) | 0 (0.0) | 0.566 | |  |
|  |  |  |  | |  |
| **SUBTOTAL Procedure** | OG (n=25) | RAMIG (n=13) | p-value | |  |
| Postoperative hospital stay (days)^c^ | 15 (12, 22.5) | 7 (6.5, 10) | <0.001^*^ | |  |
| ICU/IMC length of stay (days)^c^ | 5 (3, 6.5) | 1 (1, 1.5) | <0.001^*^ | |  |
| Surgical duration (hh:mm) ^c^ | 4:14 (3:08, 5:24) | 7:03 (5:12, 8:02) | 0.001^*^ | |  |
| UICC grouped^a^ |  |  | 0.689 | |  |
| 0-II^a^ | 20 (80.0) | 9 (69.2) |  | |  |
| III-IV^a^ | 5 (20.0) | 4 (30.8) |  | |  |
| Pathological Nodal Status^b^ | 0 (0, 0.75) | 0.5 (0.0, 2.75 | 0.212 | |  |
| Positive lymph nodes^c^ | 0 (0, 0.75) | 1 (0.0,6.75) | 0.177 | |  |
| Harvested lymph nodes^c^ | 17.5 (12, 26.75) | 29 (21, 32.75) | 0.022^*^ | |  |
| NRS rest PRE^c^ | 0 (0,0) | 0 (0,0) | 0.585 | |  |
| NRS rest POD 1^c^ | 1.5 (1,2.25) | 2 (0.75, 2.75) | 1.0 | |  |
| NRS rest POD 3^c^ | 1 (0,2) | 1 (0, 2.5) | 0.937 | |  |
| NRS rest POD 5^c^ | 1.5 (0.75, 2.5) | 0.5 (0.125, 1) | 0.049^*^ | |  |
| NRS rest POD 7^c^ | 1 (0,2) | 0.5 (0, 2) | 0.662 | |  |
| NRS activity PRE^c^ | 0 (0,0) | 0 (0,0) | 0.927 | |  |
| NRS activity POD 1^c^ | 3 (2, 4) | 3 (2, 3.88) | 0.860 | |  |
| NRS activity POD 3^c^ | 2.5 (1, 3.5) | 1.5 (1, 2.5) | 0.220 | |  |
| NRS activity POD 5^c^ | 3 (2, 4) | 1.5 (0.63, 2.75) | 0.021^*^ | |  |
| NRS activity POD 7^c^ | 2 (0,3) | 0.75 (0, 2.75 | 0.457 | |  |
| Total MME (mg)^c^ | 200 (105, 350) | 110 (59, 205) | 0.064 | |  |
| MME per kg body weight (mg/kg)^c^ | 2.88 (1.50, 5.12) | 1.16 (0.87, 3.06) | 0.028^*^ | |  |
| MME per kg per POD (mg/kg/day)^c^ | 0.18 (0.11, 0.26) | 0.17 (0.13, 0.28) | 0.903 | |  |
| Blood loss (mL)^c^ | 200 (50, 300) | 200 (50, 325) | 0.649 | |  |
| Opioid-free patients ^a^ | 0 (0.0) | 1 (7.7) | 0.342 | |  |
| Severe complications  (MCDC ≥ 3b)^a^ | 3 (12.0) | 0 (0.0) | 0.538 | |  |
| Pneumonia^a^ | 1 (4.0) | 1 (7.7) | 1.0 | |  |
| Anastomotic leakage^a^ | 4 (16.0) | 0 (0.0) | 0.278 | |  |
| Reoperation^a^ | 3 (12.0) | 0 (0.0) | 0.538 | |  |
| Hospital mortality^a^ | 1 (4.0) | 0 (0.0) | 1.0 | |  |
| Pancreatic fistula^a^ | 0 (0.0) | 0 (0.0) | - | |  |
|  |  |  | |  | |

^a^ Data are presented as absolute numbers and percentages; statistical comparison performed using Fisher’s exact test (nominal variables).

^b^ Data are presented as absolute numbers and percentages; statistical comparison performed using the MWU test (ordinal variables).

^c^ Data are presented as median and interquartile range (IQR); statistical comparison performed using the MWU test (continuous variables).

^*^ p < 0.05 was considered statistically significant.

Abbreviations: ICU/IMC, Intensive Care Unit/Intermediate Care; UICC, Union for International Cancer Control; MCDC, Modified Clavien-Dindo Classification; MME, Morphine Milligram Equivalent; POD, Postoperative Day; NRS, Numerical Rating Scale; PRE, Preoperative; MWU, Mann-Whitney U; IQR, interquartile range.
